# Breathtaking heights: Lung mechanics and pulmonary extravascular fluid accumulation in female climbers during the K2 expedition

**DOI:** 10.1113/EP093452

**Published:** 2026-02-16

**Authors:** Guia Tagliapietra, Pasquale Pio Pompilio, Chiara Veneroni, Davide Bizzotto, Raffaele Dellacà, Giacomo Strapazzon, Giovanni Vinetti, Lorenza Pratali, Annalisa Cogo, The K2‐70 Operation Study Group

**Affiliations:** ^1^ Institute of Sport Sciences, Faculty of Biology and Medicine University of Lausanne Lausanne Switzerland; ^2^ Società Italiana di Medicina di Montagna (SIMeM) Padova Italy; ^3^ Restech SRL Milan Italy; ^4^ Department of Electronics, Information and Biomedical Engineering (DEIB) Politecnico di Milano University Milan Italy; ^5^ Mountain Clinic and Institute of Mountain Emergency Medicine Eurac Research Bolzano Italy; ^6^ Institute of Clinical Physiology, Department of Biomedical Sciences National Research Council Rome Italy; ^7^ Exercise and Sport Science Center University of Ferrara Ferrara Italy

**Keywords:** acclimatization, B‐lines, exercise, field study, forced oscillation technique, lung interstitial oedema, pulmonary artery pressure, respiratory reactance, very high terrestrial altitude

## Abstract

Female physiological responses to hypoxia remain underexplored, despite growing participation in mountaineering expeditions. Pulmonary interstitial oedema described during acute hypobaric hypoxia can be exacerbated by prolonged physical exercise. It is unclear whether these factors alter the mechanical properties of the respiratory system. We tested the hypothesis that prolonged exercise at terrestrial high altitude would result in interstitial oedema, quantified as B‐lines by lung ultrasound, decreased respiratory reactance (*X*_rs_) and lower respiratory resistance (*R*_rs_), probably owing to reduced air density. Seven women, participating in the 2024 K2 female expedition, underwent respiratory oscillometry at Skardu (2228 m), Goro II (4319 m) and K2 Base Camp (5100 m) at 6 h post‐arrival (K2BC1), after 24 h (K2BC2) and post‐descent from camp I–II (K2BCPC; 6060 and 6654 m). The B‐lines and systolic pulmonary artery pressure were assessed at Skardu, K2BC2 and K2BCPC. The inspiratory component of *X*_rs_ became more negative with increasing altitude (e.g., Skardu vs. K2BC1, −1.42 ± 0.25 vs. −1.62 ± 0.27 cmH_2_O·s/L; *P* = 0.003), indicating reduced lung compliance. Both B‐lines (1.0 ± 1.4 vs. 11.9 ± 9.3; *P* = 0.039) and systolic pulmonary artery pressure (21.9 ± 6.7 vs. 33.0 ± 2.7 mmHg; *P* < 0.001) increased significantly from Skardu to K2BCPC. The inspiratory component of *R*_rs_ decreased from Skardu to K2BC2 (2.88 ± 0.69 vs. 2.34 ± 0.82 cmH_2_O·s/L; *P* = 0.003). In women, prolonged exercise at high altitude promotes the development of interstitial oedema (shown by the increase in B‐lines), which is accompanied by a reduction in inspiratory *X*_rs_, indicative of increased lung stiffness.

## Abstract (highlights)

- **What is the central question of this study?**
  Many climbers can develop subclinical pulmonary interstitial oedema at high altitude. However, the effect of interstitial oedema on respiratory mechanics is little studied and has never been studied in women. Female physiological responses to hypoxia continue to be underexplored.
- **What is the main finding and its importance?**
  In an all‐female expedition to K2, we found that prolonged exercise at terrestrial high altitude decreased respiratory reactance and increased systolic pulmonary artery pressure and lung ultrasound B‐lines, reflecting formation of interstitial oedema, which made the lung parenchyma stiffer. These results help us to understand better the pulmonary responses to severe hypoxia and exercise in women.

## 1 INTRODUCTION

At high altitude, reduced oxygen availability triggers protective responses among unacclimatized lowlanders, including increased ventilation, heart rate (HR) and cardiac output, in addition to pulmonary vasoconstriction to maintain adequate oxygen delivery and tissue perfusion (Mallet et al., 2023). A rise in pulmonary artery pressure has also been observed during exposure to hypobaric hypoxia (Bouzat et al., 2013; Maggiorini et al., 2001; Pratali et al., 2010).

Accumulating evidence suggests that hypoxia can lead to the development of subclinical pulmonary interstitial oedema (IO), probably attributable to enhanced endothelial permeability and impaired extravascular fluid clearance, with a potential contribution of increased capillary pressure (Bouzat et al., 2013; Cremona et al., 2002; Miserocchi, 2025; Pratali et al., 2010). The accumulation of extravascular fluid in the lungs is reflected by an increase in lung ultrasound B‐lines (Bouzat et al., 2013; Pratali et al., 2010; Strapazzon et al., 2015) and can impair lung mechanics (Cogo & Miserocchi, 2011). Another factor that can promote IO formation even at sea level is intense or prolonged physical exercise (Miserocchi & Beretta, 2023), potentially further affecting lung mechanics.

Regarding the effects of high altitude on lung compliance, some investigations have reported a reduction in respiratory reactance (*X*_rs_) measured at 5 Hz, and in static and dynamic lung compliances after 2 days at 4559 m (Pellegrino et al., 2010). Conversely, other studies observed no significant changes in lung compliance (Dehnert et al., 2010), with a decrease only in lung elastic recoil at 3457 m (Gautier et al., 1982). Overall, and based on the reported studies, the results remain mixed, probably reflecting differences in altitude, acclimatization status, exercise intensity and measurement techniques used.

Furthermore, the lower temperature and humidity of mountain climates cause hyperventilation of cold and dry air, particularly during exercise, which can trigger airway inflammation, epithelial injury, airway hyper‐responsiveness and asthma attacks (Cogo, 2011; Hanstock et al., 2020). Although cold, dry air and increased ventilatory drive tend to raise airway resistance (*R*_rs_), reduced air density at high altitude exerts an opposite effect, lowering *R*_rs_ and facilitating airflow (Cogo et al., 1997; Gautier et al., 1982).

Although an increasing number of women reach high altitude for leisure, sport, professional tasks and participation in expeditions, they continue to be underrepresented in research exploring adaptive responses to hypoxia (Horakova et al., 2023). However, anatomical and physiological differences between the sexes make the results obtained in men not directly applicable to women (Burtscher et al., 2024; Raberin et al., 2024). Investigations on IO at high altitude have also predominantly involved male participants; only one study provided evidence for an effect of biological sex on extravascular fluid accumulation (Strapazzon et al., 2015). Nevertheless, the study did not simultaneously assess lung mechanics. As a result, it remains unclear whether the development of IO is accompanied by impairments in lung mechanics among women. Experimental work lends support to this hypothesis. Findings from a rat model indicate that *X*_rs_, measured by respiratory oscillometry, decreases in the presence of experimentally induced IO by intravenous saline infusion, suggesting that its monitoring could serve as a potential early marker for development of pulmonary oedema in clinical practice (Dellacà et al., 2008). To the best of our knowledge, no studies have explored the combined effect of high and very high altitude and prolonged physical exercise on pulmonary mechanics, using respiratory oscillometry, together with lung ultrasound B‐line scores, in women on the way up to an 8000 m peak. The appearance of portable respiratory oscillometry devices, together with the possibility of performing lung ultrasound using hand‐held echography systems, provides a reliable approach to address this gap (Kaminsky et al., 2022; Picano & Pellikka, 2016; Veneroni et al., 2024; Wooten et al., 2019).

The aim of this investigation was to assess the impact of participating in an expedition to K2 on lung mechanics in women, using a within‐subject study design. We hypothesized that prolonged exposure to hypoxia and the exercise carried out during ascent could lead to the development of IO, with consequent alterations of respiratory mechanics.

## 2 MATERIALS AND METHODS

### 2.1 Participants

Eight female climbers (four Italians and four Pakistani) participating in the K2‐70 women expedition during the summer of 2024 were enrolled in this study. In the group, three Italians and two Pakistani were mountain guides, and the other climbers either lived at ∼2200 m or were frequently exposed to very high altitudes. Inclusion criteria required participants to be ≥18 years old and in good health.

Antropometric characteristics were as follows: age range, 18–52 years; average height, 162 ± 7 cm; body mass, 57 ± 4 kg; and body mass index, 21.7 ± 2.6 kg/m^2^.

Ethical approval was obtained from the Ethics Committee for Clinical Trials of the Autonomous Province of Bolzano no. 5–2024. All participants provided written informed consent prior to their voluntary involvement. The study was conducted in accordance with the latest guidelines of the *Declaration of Helsinki*.

### 2.2 Study design

**FIGURE 1:**
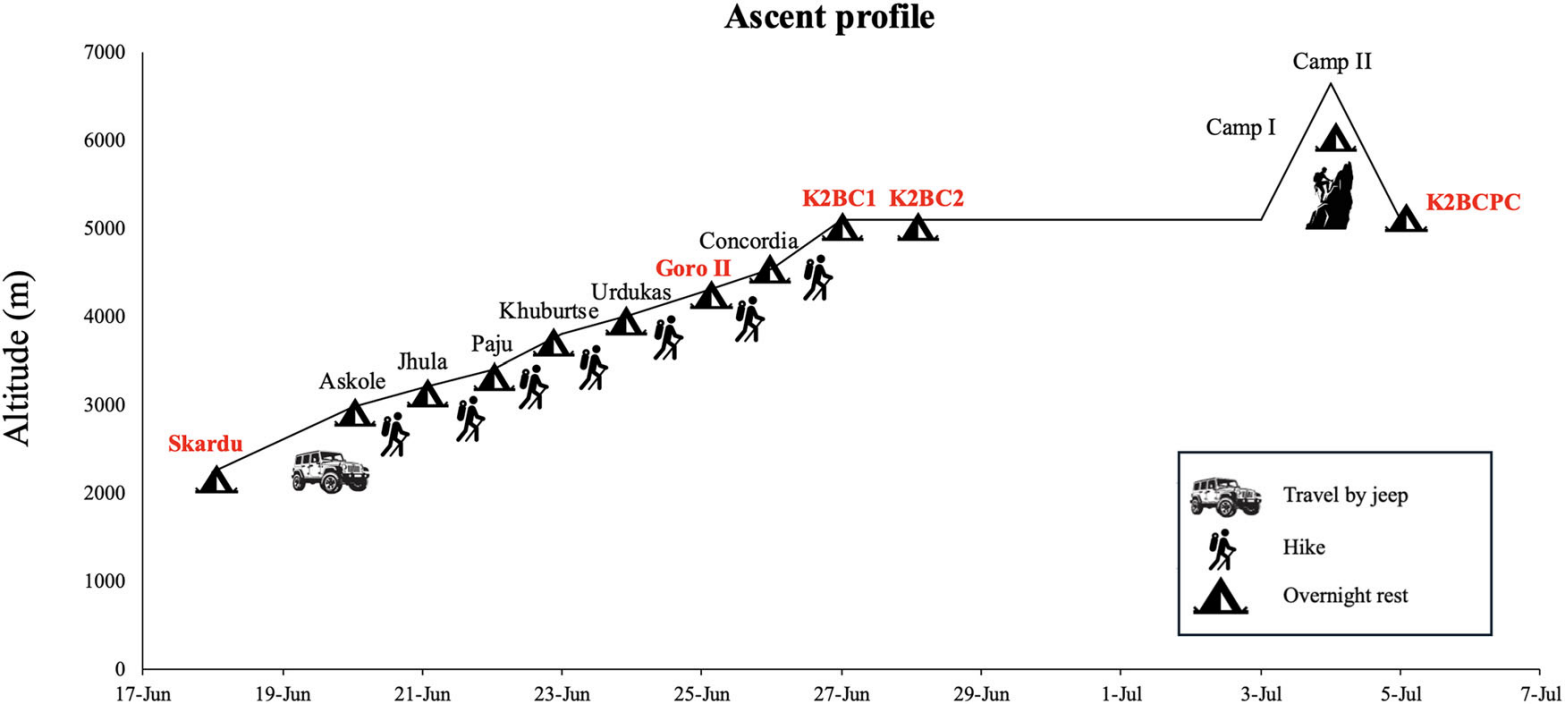
Graphical representation of the ascent profile from Skardu to K2 base camp. Abbreviations: K2BC1 and K2BC2 represent the first and second day at K2 base camp, respectively; K2BCPC indicates measurements performed at base camp post‐descent from camp I and II. Red text denotes locations where physiological assessments were conducted.

The ascent profile is detailed in Figure 1. In June 2024, the climbers reached Skardu (2228 m, Pakistan) by aeroplane and car, and they underwent pulmonary and cardiovascular assessments at rest. Then, climbers travelled (for 8 h) by jeep to Askole (2980 m) and, after a 5 day trek, reached Goro II (4319 m; total positive elevation gain 1339 m, mean positive elevation gain/day 267.8 m), where lung mechanics and other resting parameters [i.e., HR and peripheral oxygen saturation ($S_{pO_{2}}$)] were assessed ∼6 h after the arrival of the group (average duration of the last day trek, 4 h 52 min ± 57 min). This standardized time window was chosen to minimize acute confounding effects of the trek and initial hypobaric hypoxic exposure, aligning with international recommendations (Roach et al., 2018).

K2 Base Camp (K2BC 5100 m) was reached after a total of 7 days of trekking. At this location, measurements were conducted ∼6 h after the arrival of the group, following a final day of trekking that lasted an average of 5 h 30 min ± 30 min (K2BC1).

Measurements were repeated after an overnight stay at the same altitude (K2BC2) and post‐descent from higher camps (K2BCPC): camp I (6060 m) and camp II (6654 m) for all climbers except one, who reached the advanced base camp (5300 m) and returned to K2BC on the same day without an overnight stay. For this reason, this climber was excluded from the analyses, and results are based on data from *n *= 7 climbers.

On average, participants ascended to camps I and II 7 ± 1 days after K2BC1. The mean duration of exercise was 8 h 50 min ± 2 h 15 min during the ascent to higher camps and 4 h 50 min ± 1 h 50 min for the descent to K2BC. Measurements at K2BCPC were performed 8 ± 1 days after K2BC1.

Ambient temperature in Skardu was 16°C, at Goro II 0°C, and at K2BC it varied from −1°C to −4°C.

Data are reported according to the STAR protocol (Brodmann Maeder et al., 2018), which outlines a set of key parameters essential for research in high‐altitude medicine (i.e., study setting, individual factors and high‐altitude illnesses).

### 2.3 Respiratory oscillometry

Lung mechanics were assessed using respiratory oscillometry. By applying small‐amplitude pressure oscillations at the mouth, this technique measures *R*_rs_, which reflects the resistive properties of the respiratory system, and *X*_rs_, indicative of the elastic and inertial characteristics of the airways (Veneroni et al., 2024). Measurements were conducted in triplicate at rest during quiet breathing, at 5 Hz, using a portable device (FIRST, RESTECH Italy), in accordance with international standards (King et al., 2020). The device was calibrated and accuracy verified on each measurement day using a mechanical test object provided with the device to ensure quality of the results. Additional tidal breathing parameters analysed included the within‐breath inspiratory (R5_insp_ and X5_insp_) and expiratory (R5_exp_ and X5_exp_) components of *R*_rs_ and *X*_rs_. *Z*‐scores were computed according to Oostveen et al. (2013). The difference between mean inspiratory and expiratory *X*_rs_ (ΔX5) was also calculated. The same device was used to collect the following breathing pattern parameters: respiratory duty cycle (*T*_insp_/*T*_tot_), respiratory rate, tidal volume, ventilation and mean inspiratory and expiratory flows. For all oscillometric acquisitions, the climbers were seated with the chin slightly elevated, wearing a nose clip, and their cheeks were supported by the same investigator (G.T.) to minimize upper airway shunt compliance. These procedures were applied in the same way at all stations. Assessments were performed inside a dedicated tent to ensure that atmospheric conditions did not affect the acquisitions. Each acquisition (including three repeated trials) required ∼7 min.

Measurements were performed at Skardu, Goro II, K2BC1, K2BC2 and K2BCPC.

### 2.4 Acute mountain sickness score

Acute mountain sickness (AMS) was assessed, after at least 6 h at the same altitude, at Skardu, Goro II, K2BC1, K2BC2 and K2BCPC using the Lake Louise AMS score (LLS) (Roach et al., 2018). A total score of at least three in the presence of headache together with at least one other symptom, i.e., gastrointestinal, fatigue and/or weakness and dizziness/light‐headedness, was considered diagnostic for AMS. The prevalence of AMS was calculated as the proportion of individuals with AMS to the total number of subjects exposed to very high altitude and expressed as a percentage.

### 2.5 Lung ultrasound B‐lines

B‐Lines reflect the development of IO, which might result from increased capillary permeability and increased intravascular hydrostatic pressure (Bouhemad et al., 2015). The presence of B‐lines was assessed on lung ultrasound at Skardu, K2BC2 and K2BCPC using a portable echography machine (Vivid I, General Electric Healthcare Clinical System) with a linear probe (10 MHz). The B‐lines were defined as discrete, vertical hyperechoic reverberation artefacts going from the pleural line to the bottom of the screen and moving simultaneously with lung sliding, as previously suggested (Volpicelli et al., 2012).

Lung ultrasound was performed as previously described on the anterior, lateral and posterior hemithorax, on both the right and left sides, relying on a previously validated score (Gargani et al., 2020). A score of (i) zero was attributed when fewer than three separated B‐lines were observed, (ii) one when at least three B‐lines or coalescent B‐lines occupying ≤50% of the screen without a clearly irregular pleural line were detected, (iii) two when coalescent B‐lines occupied >50% of the screen without a clearly irregular pleural line and (iv) three reflecting large consolidations (≥1 cm). The final score was obtained by summing the scores of each of the 16 areas. Climbers were supine during the anterolateral scanning and sitting during the posterior scanning.

Measurements were performed at Skardu, K2BC2 and K2BCPC.

### 2.6 Transthoracic echocardiography

Echocardiography was performed using a portable echography system (Vivid I, General Electric Healthcare Clinical System) with a cardiac probe (3.5 MHz) and systolic pulmonary artery pressure (PAPs) was assessed from the maximum velocity of tricuspid regurgitation, according to the most recent American and European Recommendations and Guidelines (Lang et al., 2015; Rudski et al., 2010). The measurements were carried out at Skardu, K2BC2 and K2BCPC.

Examinations were performed in a dedicated medical tent to ensure that atmospheric conditions did not influence the acquisitions. The important feature of lung ultrasound is that it can be performed in any setting and in a wide range of environmental conditions using even small portable echocardiographs (Otto et al., 2009; Pratali et al., 2010).

### 2.7 Resting measurements

Brachial systolic blood pressure (SBP) and diastolic blood pressure (DBP) were measured in a seated position at Skardu, K2BC2 and K2BCPC using an automated device (OMRON‐705IT; Omron, Kyoto, Japan). HR and finger $S_{pO_{2}}$ were recorded with a pulse oximet (Ohmeda TuffSat; GE Healthcare, Helsinki, Finland) after warming the fingers identically at all locations (i.e., Skardu, Goro II, K2BC1, K2BC2 and K2BCPC).

All the reported values represent the average of three consecutive measurements taken ≥2 min apart.

### 2.8 Statistical analysis

*Post hoc* comparisons with Bonferroni correction were used to explore specific differences between altitude levels when a significant effect (*P* < 0.05) was detected using repeated‐measures ANOVA. When residuals were not normally distributed, the Friedman test was used instead, followed by Conover's *post hoc* test with Bonferroni correction when a significant effect was detected. Because one participant stopped at the advanced base camp, these analyses were performed on data from seven climbers. After verifying the assumptions of normality, linearity and homoscedasticity of residuals, a simple linear regression was used to evaluate the relationship between X5_insp_ and ultrasound B‐lines.

Furthermore, to determine whether there was a statistically significant difference in AMS prevalence across multiple altitude locations, Cochran's *Q* test was applied to data from the same seven subjects. Spearman's correlations were used to assess the relationships between ΔX5, B‐lines, PAPs, LLS and $S_{pO_{2}}$. The significance level for all tests was set at *P* < 0.05. All statistical analyses were performed in R (v.4.4.2). Data are presented as the mean ± SD. Qualitative variables are reported as *n* (%).

## 3 RESULTS

### 3.1 Respiratory oscillometry

Respiratory oscillometry was feasible in all subjects (feasibility 100%). Respiratory resistance (R5) and reactance (X5), their within‐breath inspiratory and expiratory components and percentage predicted values, measured at different altitudes, are displayed in Table 1. *Post hoc* within‐subject Bonferroni‐corrected pairwise comparisons revealed a significant decrease in R5_insp_ from Skardu to K2BC2 (*P* = 0.003) and K2BCPC (*P* < 0.001). R5_exp_ also showed a significant reduction from Skardu to K2BC2 (*P* = 0.008) and K2BCPC (*P* = 0.023). As a result, R5_tot_ was significantly higher at Skardu than at K2BC2 and K2BCPC (*P* = 0.014 for both time points), indicating reduced respiratory resistance with increasing altitude. Significant reductions were also observed in X5_insp_ from Skardu to Goro II (*P* = 0.006) and K2BC1 (*P* = 0.003), indicating that peripheral lung tissue had reduced compliance or was compressed at altitude. This decline in the inspiratory component of the reactance contributed to an overall decrease in X5_tot_ from Skardu to K2BC1 (*P* = 0.018), whereas X5_exp_ did not differ significantly across conditions.

**TABLE 1:** Lung mechanics and ventilatory parameters at different altitude levels.

Parameter	Skardu	Goro II	K2BC1	K2BC2	K2BCPC	*P*‐value
R5_insp_, cmH_2_O·s/L	2.88 ± 0.69	2.53 ± 0.60	2.56 ± 0.68	2.34 ± 0.82**	2.25 ± 0.54***^†^	**0.003**
R5_exp_, cmH_2_O·s/L	3.33 ± 1.11	2.97 ± 0.74	3.17 ± 0.97	2.79 ± 0.90**	2.85 ± 1.14*	**0.012**
R5_tot_, cmH_2_O·s/L	3.16 ± 0.95	2.81 ± 0.68	2.93 ± 0.84	2.62 ± 0.85*	2.61 ± 0.89*	**0.012**
R5_tot_, *z*‐score	0.417 ± 1.120	0.400 ± 0.962	0.156 ± 1.110	−0.296 ± 1.280	−0.314 ± 1.270*	**0.014**
X5_insp_, cmH_2_O·s/L	−1.42 ± 0.25	−1.60 ± 0.27**	−1.62 ± 0.27**	−1.56 ± 0.22	−1.49 ± 0.24	**0.008**
X5_exp_, cmH_2_O·s/L	−1.01 ± 0.23	−1.11 ± 0.19	−1.23 ± 0.32	−1.13 ± 0.15	−1.03 ± 0.24	0.105
X5_tot_, cmH_2_O·s/L	−1.17 ± 0.23	−1.30 ± 0.22	−1.39 ± 0.29*	−1.29 ± 0.18	−1.21 ± 0.23	**0.040**
X5_insp_, *z*‐score	−0.949 ± 0.548	−1.380 ± 0.540***	−1.410 ± 0.627**	−1.300 ± 0.537	−1.140 ± 0.463	**<0.001**
X5_tot_, *z*‐score	−0.379 ± 0.598	−0.701 ± 0.499	−0.900 ± 0.744*	−0.704 ± 0.485	−0.503 ± 0.554	**0.039**
ΔX5, cmH_2_O·s/L	−0.404 ± 0.126	−0.493 ± 0.136	−0.386 ± 0.131	−0.437 ± 0.101	−0.462 ± 0.141	0.216
*T*_insp_/*T*_tot_	0.374 ± 0.029	0.384 ± 0.031	0.402 ± 0.032	0.384 ± 0.036	0.391 ± 0.025	0.070
Respiratory rate, breaths/min	14.1 ± 4.6	15.5 ± 4.8	16.2 ± 3.9	16.0 ± 4.2	17.2 ± 3.7*	**0.030**
Tidal volume, L	0.73 ± 0.52	0.82 ± 0.51*	0.89 ± 0.61**	0.95 ± 0.66***	0.83 ± 0.54**	**0.001**
Ventilation, L/min	8.8 ± 2.3	11.1 ± 2.8	12.8 ± 4.3***	13.2 ± 3.6***	12.9 ± 4.0***	**0.001**
Mean inspiratory flow, L/s	0.39 ± 0.10	0.49 ± 0.15	0.54 ± 0.21**	0.58 ± 0.18***	0.54 ± 0.18*	**0.003**
Mean expiratory flow, L/s	−0.24 ± 0.06	−0.30 ± 0.06	−0.36 ± 0.10***^#^	−0.36 ± 0.09***^#^	−0.35 ± 0.11***^#^	**<0.001**

*Note*: Values are the mean ± SD. The *z*‐scores were computed from Oostveen et al. (2013). *n* = 7. Abbreviations: K2BC1 and K2BC2, the first and second day at K2 base camp, respectively; K2BCPC, post‐descent from camp I and II to base camp; R5, respiratory resistance at 5 Hz; R5_exp_, within‐breath expiratory component of respiratory resistance at 5 Hz; R5_insp_, within‐breath inspiratory component of respiratory resistance at 5 Hz; *T*_insp_/*T*_tot_, ratio between inspiratory time and total breath duration; X5, respiratory reactance at 5 Hz; X5_exp_, within‐breath expiratory component of respiratory reactance at 5 Hz; X5_insp_, within‐breath inspiratory component of respiratory reactance at 5; ΔX5, the difference between mean inspiratory and expiratory reactance at 5 Hz.

Statistical significance from pairwise comparisons with Bonferroni correction is displayed in bold for clarity and indicated as follows: *P < 0.05, ** *P* < 0.01 and *** *P* < 0.001, representing significant differences from Skardu; ^#^ *P* < 0.05, representing significant differences from Goro II; and **^†^** *P* < 0.05, representing significant differences from K2BC1.

### 3.2 Ventilation and breathing pattern

Ventilation rose significantly from Skardu to K2BC1 (*P* < 0.001), mainly due to a significantly greater tidal volume (*P* = 0.002), whereas respiratory rate did not reach statistical significance (*P* = 0.064). Expiratory time was longer at Skardu (3.05 ± 1.23 s) compared with K2BC1 (2.41 ± 0.81 s; *P* = 0.016), K2BC2 (2.55 ± 0.95 s; *P* = 0.043) and K2BCPC (2.25 ± 0.61 s; *P* = 0.009), whereas the duration of the inspiratory phase did not exhibit significant changes (*P* = 0.126). Both the mean inspiratory and expiratory flows significantly increased with altitude (e.g., maximal inspiratory flow at Skardu vs. K2BC1; *P* = 0.003; other values are shown in Table 1).

### 3.3 Ultrasound B‐lines and peripheral oxygen saturation

The B‐line scores showed a marked and progressive increase from Skardu to K2BC2 (*P* = 0.039) and K2BCPC (*P* = 0.039), suggesting a progressive accumulation of extravascular fluid in the lungs, consistent with mild but evolving IO (Table 2).

**TABLE 2:** Lung ultrasound B‐lines and peripheral oxygen saturation at different altitudes levels.

Parameter	Skardu	Goro II	K2BC1	K2BC2	K2BCPC	*P*‐value
B‐lines	1.0 ± 1.4	–	–	7.9 ± 4.9*	11.9 ± 9.3*	**0.013**
Peripheral oxygen saturation, %	95.0 ± 1.2	89.0 ± 3.3**	84.4 ± 4.3**^##^	85.9 ± 3.13***^#^	85.3 ± 3.5***^#^	**<0.001**

*Note*: Values are the mean ± SD. Cells with dashes indicate that the respective measurements were not conducted at those time points. *n* = 7. Abbreviations: K2BC1 and K2BC2, the first and second day at K2 base camp, respectively; K2BCPC, post‐descent from camp I and II to base camp.

Statistical significance from pairwise comparisons with Bonferroni correction is displayed in bold for clarity and indicated as follows: *P < 0.05, ** *P* < 0.01 and *** *P* < 0.001, representing significant differences from Skardu; and ^#^ *P* < 0.05 and ^##^ *P* < 0.01, representing significant differences from Goro II.

The $S_{pO_{2}}$ decreased significantly from Skardu to K2BC1 (*P* = 0.002), K2BC2 (*P* < 0.001) and K2BCPC (*P* < 0.001).

**FIGURE 2:**
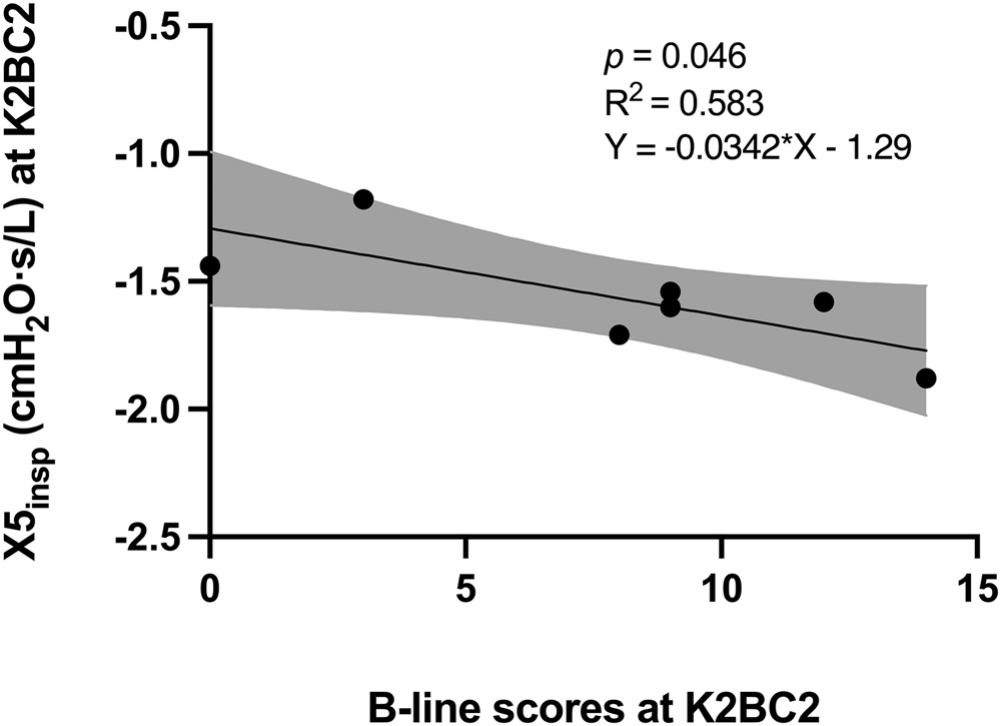
Simple linear regression between B‐lines and inspiratory reactance (X5_insp_) at K2 base camp on day 2 (K2BC2). Each dot represents a participant. The continuous line indicates the best‐fitting regression line, and the shaded area represents the 95% confidence interval. *n* = 7.

Linear regression analysis showed that B‐line scores were significantly associated with more negative X5_insp_ at K2BC2 (β = −0.034; *P* = 0.046; *R* ^2^ = 0.58; Figure 2), indicating a moderately strong relationship between IO and lung compliance.

### 3.4 Haemodynamics

The PAPs was significantly higher at K2BC2 (*P* = 0.033) and K2BCPC (*P* < 0.001) than at Skardu (Table 3).

**TABLE 3:** Echocardiographic and haemodynamic measurements at different altitude levels.

Parameter	Skardu	Goro II	K2BC1	K2BC2	K2BCPC	*P*‐value
PAPs, mmHg	21.9 ± 6.7	–	–	29.4 ± 5.2*	33.0 ± 2.7***	**0.007**
Peripheral oxygen saturation, %	95.0 ± 1.2	89.0 ± 3.3**	84.4 ± 4.3**^##^	85.9 ± 3.13***^#^	85.3 ± 3.5***^#^	**<0.001**
Heart rate, beats/min	63.4 ± 9.0	84.4 ± 10.5**	85.0 ± 10.6***	83.4 ± 10.1**	85.9 ± 16.4	**<0.001**
Systolic blood pressure, mmHg	107 ± 6	–	–	123 ± 7**	118 ± 9	**0.001**
Diastolic blood pressure, mmHg	66.1 ± 6.7	–	–	80.3 ± 5.6**	79.6 ± 8.6*	**<0.001**

*Note*: Values are the mean ± SD. Cells with dashes indicate that the respective measurements were not conducted at those time points. *n* = 7. Abbreviations: K2BC1 and K2BC2, the first and second day at K2 base camp, respectively; K2BCPC, post‐descent from camp I and II to base camp; PAPs, systolic pulmonary artery pressure.

Statistical significance from pairwise comparisons with Bonferroni correction is displayed in bold for clarity and indicated as follows: *P < 0.05, ** *P* < 0.01 and *** *P* < 0.001, representing significant differences from Skardu; and ^#^ *P* < 0.05 and ^##^ *P* < 0.01, representing significant differences from Goro II.

As expected, HR rose from Skardu to Goro II (*P* = 0.003), K2BC1 (*P* < 0.001) and K2BC2 (*P* = 0.005). Both DBP (*P* = 0.004) and SBP (*P* = 0.002) exhibited a significant increase from Skardu to K2BC2, as indicated in Table 3.

### 3.5 Relationship between B‐lines, ΔX5 and PAPs

**FIGURE 3:**
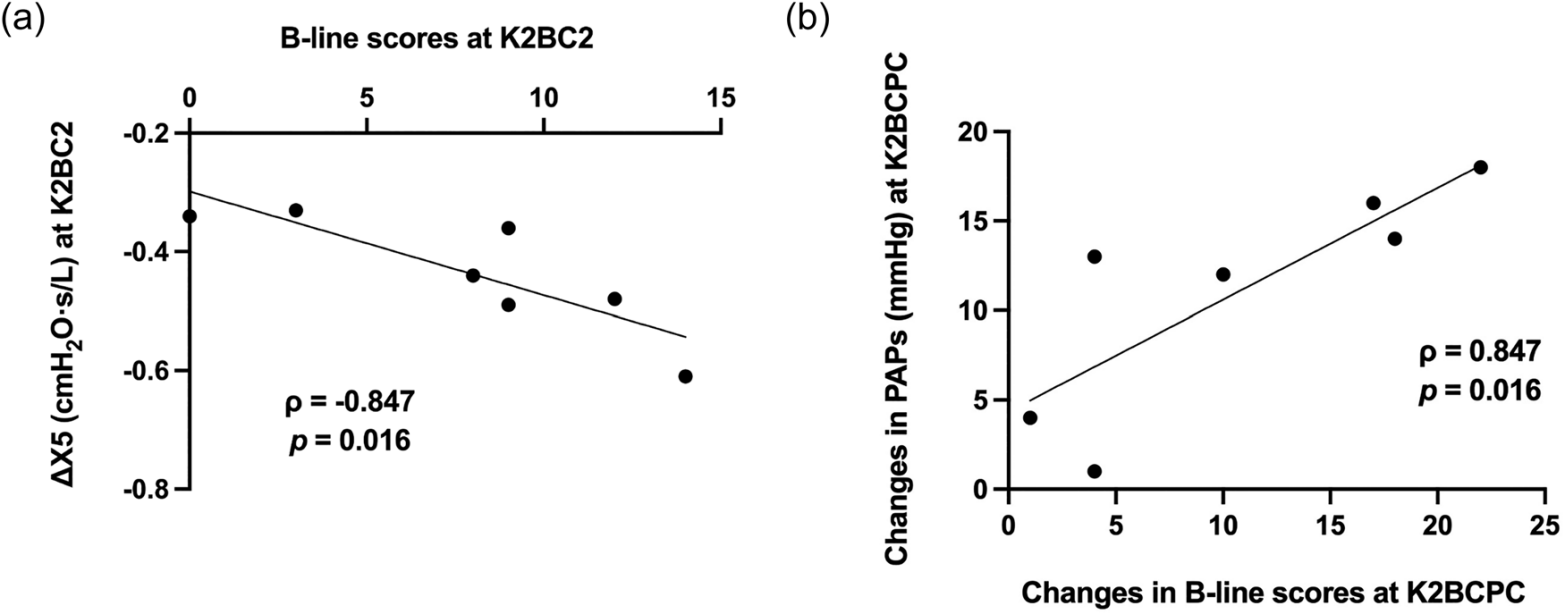
Relationship between B‐line scores and the ΔX5 at K2 base camp on day 2 (K2BC2) (a) and between changes in B‐line scores and systolic pulmonary artery pressure (PAPs) from Skardu to post‐descent from higher camps (K2BCPC) (b). *n = 7*.

At K2BC2, a significant negative correlation was observed between B‐line scores and the ΔX5 (ρ = −0.847; *P* = 0.016; Figure 3a). A significant positive correlation was found between changes in B‐lines and in PAPs from Skardu to K2BCPC (ρ = 0.847; *P* = 0.016; Figure 3b). These associations suggest that greater extravascular fluid accumulation in the lungs was linked to both a lower ΔX5 and larger increases in PAPs.

### 3.6 Lake Louise score

Up to K2BC, LLS was less than three in all climbers, whereas at K2BCPC 57% of them developed symptoms of AMS, resulting in a significant difference in AMS prevalence across time points (*P* = 0.003). Spearman's correlations revealed a significant positive relationship between the ΔX5 at K2BC1 and LLS at K2BCPC (ρ = 0.891; *P* = 0.007). As expected, at K2BCPC, lower $S_{pO_{2}}$ values were strongly associated with higher LLS (ρ = −0.955; *P* < 0.001).

## 4 DISCUSSION

The main new finding of this study is that, during ascent to very high altitude, X5_insp_ became more negative in female climbers, indicating reduced lung compliance, while lung ultrasound B lines, reflecting the development of IO, and PAPs increased. Moreover, respiratory oscillometry has been shown to be time efficient and feasible, even in extreme environments beyond traditional indoor clinical or laboratory settings.

Multiple studies have suggested that most climbers might develop subclinical IO (Bouzat et al., 2013; Cogo & Miserocchi, 2011; Cremona et al., 2002; Pratali et al., 2010; Strapazzon et al., 2015) during an ascent and a sojourn at high altitude, as indicated both indirectly, through respiratory function tests, and directly, through imaging. To our knowledge, this is the first study to analyse respiratory mechanics, lung ultrasound data and PAPs simultaneously, evaluated by echocardiography in an all‐female expedition to K2.

The first to report alterations in respiratory mechanics at altitude were Jaeger et al. (1979). In a cohort of male soldiers, they observed a significant decrease in transthoracic electrical impedance from low to high altitude and a subsequent increase in closing capacity. These findings were consistent with immediate changes in thoracic intravascular fluid volume and a gradual rise in extravascular fluid volume in the peribronchial spaces. However, no corresponding evidence of pulmonary oedema was detected on chest radiographs, suggesting that the latter method was not the most sensitive to detect IO. More recently, lung ultrasound has been shown to be more sensitive in non‐invasive detection of an early accumulation of extravascular fluid in the lungs (Wooten et al., 2019).

In line with previous findings, our results support the hypothesis that ascent to and permanence at very high altitude induce an increase in PAPs and IO formation (Bouzat et al., 2013; Pratali et al., 2010). The increase in B‐line scores measured by lung ultrasound, accompanied by significant reversible alterations in *X*_rs_, supports the hypothesis of peripheral airway impairments and increased parenchymal stiffness. In our study, within‐breath analyses revealed that the decrease was significant for X5_insp_, but not for X5_exp_, suggesting involvement of the pulmonary interstitium and decreased compliance, probably attributable to the development of IO. Notably, previous investigations have observed that a distinguishable characteristic of interstitial lung disease patients is that X5_insp_ is more reduced than X5_exp_, likely because the peripheral lung tissue has decreased lung compliance or is compressed (Sugiyama et al., 2013). Therefore, monitoring within‐breath inspiratory and expiratory components of *X*_rs_ might potentially serve as a specific indicator of developing IO, although confirmation in larger studies is warranted.

Regarding AMS, no symptoms were reported during the initial ascent to base camp. However, following an acclimatization climb to camp I (6060 m) and II (6654 m), more than half of the climbers (57%) developed symptoms. Notably, ΔX5 measured upon arrival at K2BC was strongly correlated with the severity of subsequent AMS symptoms. Although these observations will need to be corroborated by larger studies, they might suggest a possible role of oscillometry in the early detection of a subsequent development of altitude sickness.

Both mean inspiratory and expiratory flows increased at very high altitudes, probably owing to the lower air density, which reduced airway *R*_rs_, facilitating airflow in and out of the lungs. Moreover, changes in breathing patterns at altitude, particularly the increase in tidal volume observed during hypobaric hypoxia, might also have contributed to the enhanced inspiratory and expiratory flows. Gautier et al. (1982) postulated that the reduction in *R*_rs_ observed at altitude might also reflect the occurrence of bronchodilatation, mediated by heightened β_2_‐adrenergic activity secondary to higher catecholamine levels.

The physiological parameters and estimated pulmonary pressure were in line with previously reported responses to high altitude (Baggish et al., 2014; Mallet et al., 2023): $S_{pO_{2}}$ progressively decreased, whereas HR, DBP, SBP and PAPs significantly increased.

### 4.1 Methodological considerations

The strength of this study lies in its within‐subject design in ecologically valid, terrestrial high‐altitude conditions, combining respiratory oscillometry, lung ultrasound and echocardiographic PAPs estimates in an exclusively female cohort, filling a major gap in sex‐specific research on pulmonary response to very high altitude.

However, some limitations must be acknowledged. Firstly, the sample size is small, due to the unique nature of an expedition‐based study and inclusion of only female mountaineers. Secondly, the findings are specific to climbers. It is possible that different changes might be observed in individuals who are less acclimatized to high altitude or live permanently at sea level. Furthermore, in line with the main aim of the present study, exploring physiological and lung mechanical responses to an expedition to K2, baseline assessments were performed in Skardu, where the team of climbers met 2 days before the beginning of the trekking. Finally, ovarian hormone concentrations were not measured owing to logistical constraints. Nonetheless, growing evidence suggests that any physiological differences between menstrual cycle phases, if present, are minimal or negligible (D'Souza et al., 2023; Tagliapietra et al., 2024).

## 5 CONCLUSION

The growing involvement of women in high‐altitude activities calls for a more comprehensive understanding of their acclimatization processes for developing tailored strategies to optimize their health and performance in extreme conditions. To the best of our knowledge, this is the first study to investigate the effects of very high altitude and prolonged exercise on the complex and dynamic relationship between cardiovascular changes and lung mechanics in an all‐female expedition. This result was also possible thanks to a recently developed portable device that can be carried easily in a backpack when trekking and allows rapid on‐site measurements.

At terrestrial very high altitude, the reduced air density decreased respiratory airway *R*_rs_. Furthermore, the stress imposed by hypobaric hypoxia and prolonged exercise decreased lung compliance and increased B‐line scores, reflecting the development of IO, and increased PAPs. These findings support the use of respiratory oscillometry as a simple, reliable, non‐invasive technique for the assessment of lung mechanics in the field at terrestrial very high altitude.

Nevertheless, further research is warranted to substantiate our findings with a larger sample size and explore whether different physiological responses exist between women and men and between subjects with different levels of training and acclimatization or subjects of different ethnicities (i.e., Italian and Pakistani women).

## AUTHOR CONTRIBUTIONS

Annalisa Cogo, Lorenza Pratali and Giacomo Strapazzon conceived the research project. Lorenza Pratali, Pasquale Pio Pompilio, Giacomo Strapazzon, Giovanni Vinetti and Guia Tagliapietra performed data collection. Annalisa Cogo, Davide Bizzotto, Lorenza Pratali, Pasquale Pio Pompilio and Guia Tagliapietra performed data treatment and analysis. Annalisa Cogo, Chiara Veneroni, Raffaele Dellacà, Pasquale Pio Pompilio, Lorenza Pratali and Guia Tagliapietra interpreted the data. Guia Tagliapietra drafted the manuscript. All authors critically revised the draft, approved the final version of the manuscript and agree to be accountable for all aspects of the work in ensuring that questions related to the accuracy or integrity of any part of the work are appropriately investigated and resolved. All persons designated as authors qualify for authorship, and all those who qualify for authorship are listed.

## CONFLICT OF INTEREST

None declared.

## Data Availability

The data that support the findings of this study are available from the corresponding author upon reasonable request.
